# A comparison of mental health legislation from diverse Commonwealth jurisdictions^☆^

**DOI:** 10.1016/j.ijlp.2009.02.006

**Published:** 2009-05

**Authors:** E.C. Fistein, A.J. Holland, I.C.H. Clare, M.J. Gunn

**Affiliations:** a Cambridge Intellectual and Developmental Disabilities Research Group, Department of Psychiatry, University of Cambridge, Douglas House, 18b Trumpington Road, Cambridge, CB2 8AH, UK; b Department of Law, University of Derby, Kedleston Road, Derby, DE22 1GB, UK

**Keywords:** Involuntary treatment, Autonomy, Human rights, Legislation

## Abstract

**Introduction:**

In the regulation of involuntary treatment, a balance must be found between duties of care and protection and the right to self-determination. Despite its shared common roots, the mental health legislation of Commonwealth countries approaches this balance in different ways. When reform is planned, lessons can be learned from the experiences of other countries.

**Method:**

Criteria for involuntary treatment used in a sample of 32 Commonwealth Mental Health Acts were compared using a framework developed from standards derived from the Universal Declaration of Human Rights. Reasons for non-compliance were considered and examples of good practice were noted. Changes in the criteria used over time and across areas with differing levels of economic development were analysed.

**Results:**

1. Widespread deviation from standards was demonstrated, suggesting that some current legislation may be inadequate for the protection of the human rights of people with mental disorders. 2. Current trends in Commonwealth mental health law reform include a move towards broad diagnostic criteria, use of capacity and treatability tests, treatment in the interests of health rather than safety, and regular reviews of treatment orders. Nevertheless, there are some striking exceptions.

**Discussion:**

Explanations for deviation from the standards include differing value perspectives underpinning approaches to balancing conflicting principles, failure to keep pace with changing attitudes to mental disorder, and variations in the resources available for providing treatment and undertaking law reform. Current good practice provides examples of ways of dealing with some of these difficulties.

## 1 Introduction

This article describes and compares mental health law from countries, which, despite economic and cultural differences, share a common legal heritage. Our study investigated whether common ethical and legal principles continue to underpin mental health legislation and examined factors associated with any diversity that was established.

Admitting adults to hospital for psychiatric treatment without their consent remains a controversial issue and challenges the very basis of modern day clinical practice—that of informed consent. However, since it is generally recognized that such action may sometimes be necessary, a legal framework is required to define, and constrain, the circumstances under which this may take place. There is a balance to be achieved between, on the one hand, having the means to respond to the needs of, and/or risks posed by, a person considered to have a “mental disorder” who is not consenting to the proposed intervention, and, on the other, the risk that the use of such legislation poses with respect to a person's rights to autonomy. The potential for the political misuse of mental health legislation, as occurred in the USSR (Chodoff, 1974), in South Africa during the apartheid era (American Psychiatric Association, 1979), and is reported to be occurring in present day China (Keukens & van Voren, 2007), also requires that such legislation is underpinned by clear ethical and legal principles that are acceptable internationally.

The requirement for informed consent is an expression of an important principle in moral philosophy, namely “respect autonomy”. Autonomy has been defined in many different ways (Dworkin, 1988) but fundamentally it means having the freedom to be self-governing. The bases of the respect autonomy principle and its justifiable limitations have been the subjects of extensive philosophical debate.

The central arguments in support of the principle are that, on the one hand, it is unreasonable to override decisions that are the product of a rational will (Kant, 1997 [1785]) and, on the other, that liberty of voluntary action is an essential component of happiness (Mill, 1998 [1869]) or is instrumental to its attainment (Brock, 1988). Several authors have argued that this principle gives rise to a strong moral claim or right to autonomy. This may be derived from a reasoned claim to equal respect for dignity alongside other members of the moral community (Darwall, 2006) or from the necessity of respecting people's interest in self-determination, in order that they may promote their own best interests (Feinberg, 1980).

In contrast, the justifications for limiting autonomy rights include the prevention of harm to the person him or herself (paternalism) or of harm to others. Many authors have argued that paternalism is only justifiable if decision-making capacity is significantly impaired such that the choice being overridden is essentially non-voluntary (McMillan, 2007). Interference to prevent harm to others is justified because interference with an assailant's autonomy preserves both the autonomy and the physical integrity of any potential victims (Feinberg, 1984; Mill, 1998 [1869]).

Beginning with the *Universal Declaration of Human Rights* (United Nations, 1948) efforts have been made to codify such moral rights. The UDHR has since been operationalised in the form of enforceable instruments such as the *European Convention on Human Rights* (Council of Europe, 1950) the *American Convention on Human Rights* (Organization of American States, 1978) and the *African (Banjul) Charter on Human and Peoples*' *Rights* (Organization of African Unity, 1982).

However, in reality, mental health legislation may not fully reflect either coherent philosophical arguments or the various Conventions and Declarations, since legislation is influenced by the specific historical, social, political, and cultural context in which it is enacted. Arguably, mental health legislation in general pays insufficient attention to the human rights of people with mental disorders (Jones, 2005). Such inattention may reflect a wide variety of factors, from shortage of the necessary economic resources in the context of the enormity of other problems (Dhanda, 2005), to the dominance within society of a particular view about the appropriate balance between the delivery of treatment and respect for autonomy (Chodoff, 1984). Not surprisingly, therefore, mental health legislation varies widely across the world (Appelbaum, 1997; Bartlett & Watchirs, 2005; Gray & O'Reilly, 2001; Zinkler & Priebe, 2002). This picture is reflected in the Commonwealth of Nations, an association of 53 countries with nearly 2 billion citizens representing a broad range of religious faiths, ethnic groups, cultures and traditions (Commonwealth Secretariat, 2007), which share the same common-law basis for legal systems. Within this association, there is a variety of statutory mental health legislation, both recently enacted (Watchirs, 2005) and long-standing (Bolis, 2002; Faunce, 2005).

In recent years, guidance has been developed to try to ensure that national mental health legislation, while reflecting the conditions of the specific country, complies with human rights instruments derived from the Universal Declaration of Human Rights. Such guidance takes the form of legal principles that should be incorporated in order to balance the prima facie right to self-determination with other considerations such as welfare and public safety.

The comparative study of mental health law reported in this article has been undertaken in response to debates within Great Britain around recent reform of the *Mental Health Act 1983* (covering England and Wales) and the *Mental Health (Scotland) Act 1984*. In Scotland, the enactment of the *Mental Health (Care and Treatment) (Scotland) Act 2003* was relatively non-contentious. However, from the start, the proposals for England and Wales gave rise to profound disagreements between the Government and the majority of professional and charitable organizations (Grounds, 2001; Szmukler & Holloway, 2000; Zigmond & Holland, 2000), which remained, at least in part, unresolved when the amending *Mental Health Act 2007* was enacted.

Central to this debate have been arguments about the ethical principles that, either implicitly, or, as recommended in the *Report of the Expert Committee* (Department of Health, 1999), explicitly inform the law. Respect for autonomy is a key issue, alongside the related concept of “decision-making capacity” and the role of the assessment of capacity in determining whether compulsory treatment can and should take place. The relevance of the concept of “decision-making capacity” to mental health law has gained increasing prominence, as a result of theoretical analysis supported by evidence from empirical studies and precedent from case law. This material is summarised below.

Research suggests that the impairments of cognition, affect and insight that can characterize serious mental disorders (Ghaemi & Pope, 1994) may affect the ability to make decisions about treatment (Murphy & Clare, 2003; Okai et al., 2007); although this is by no means inevitable (Jacob et al., 2005; Wong, Clare, Holland, Watson, & Gunn, 2000). Moreover, physical disorders have also been found to compromise decision-making capacity (Raymont et al., 2004).

When considering authorization of treatment for physical illness in the absence of consent, the courts have limited their powers to cases where it is necessary to enable provision of treatment for people who lack the ability to make autonomous treatment decisions. In one of the leading cases prior to the Mental Capacity Act 2005, Re C (adult: refusal of medical treatment) [1994] 1 All E R 819 (see also the Court of Appeal decision in Re MB (adult: medical treatment) [1997] 38 BMLR 175), neither status (such as diagnosis of mental disorder) nor adverse outcome (such as the likely death of the patient) were considered sufficient justification for the infringement of a person's autonomy by imposing treatment without consent.

The British Government were advised that adults' decision-making capacity should be the pivotal issue in determining whether substitute decision-making was appropriate, regardless of whether the treatment was for a mental or physical disorder (Department of Health, 1999). In Scotland this is now the case; both the *Adults with Incapacity (Scotland) Act 2000* (regulating treatment for physical illness) and the *Mental Health (Care and Treatment) (Scotland) Act 2003* (regulating treatment for mental disorder) include impaired decision-making capacity or judgment as a criterion for non-consensual treatment. However, in England and Wales the pivotal role of capacity applies only to the non-consensual treatment of physical disorder (regulated by the *Mental Capacity Act 2005*), not to mental disorders (regulated from November 2008 by the *Mental Health Acts 1983* and *2007*).

The aims of this study were as follows: a) to describe existing mental health legislation in force in Commonwealth countries and to compare this against standards derived by two cross-national bodies from the Universal Declaration of Human Rights: b) to investigate differences that exist between the mental health legislation in different countries and consider why they may have arisen; and c) to seek to identify core principles and procedures common to the legislation of different jurisdictions.

## 2 Methods

### 2.1 Developing a framework

To enable a systematic comparison of statutory legislation to be carried out, a multi-axial framework was developed from two consensus statements regarding the regulation of involuntary psychiatric treatment: the WHO Mental Health Policy and Service Guidance Package–Mental Health Legislation & Human Rights (World Health Organization, 2003) and REC (2004)10 of the Council of Europe (Council of Europe, 2004). The guidance in these statements, which are summarised in Table 1, were both developed following extensive consultation. They demonstrate different approaches to the goal of protecting the human rights of people with mental disorders. The WHO guidelines, drawing on United Nations Resolution 46/119 (United Nations, 1991), which is, in turn, derived from the UDHR, propose four key principles for legitimate involuntary treatment: a review process must be in place, a diagnostic threshold must be passed, and a risk threshold should be set, which is variable according to whether an incapacity threshold is passed. Somewhat different are the Council of Europe (CE) recommendations, based on the interpretation of instruments such as the European Convention on Human Rights (also derived from the UDHR) and the *Convention on Human Rights and Biomedicine* (Council of Europe, 1997); they incorporate a review process, a diagnostic threshold, a therapeutic aim, and a fixed risk threshold.

It should be borne in mind that most of the legislation analysed in this study was enacted before these two guidance statements were published, and, in any case, that of the Council of Europe is legally applicable only to those jurisidictions that are members. We have incorporated both statements into the framework developed in this study with the aim of accommodating legitimate differences in approach towards the protection of human rights—including the rights of a person with mental disorder to self-determination and healthcare and the rights of others to self-determination and bodily integrity.

The framework's axes were derived from the five legal tests in the guidance provided by the two statements. Along each axis, possible approaches to framing the test, derived from a pilot review of a sample of current mental health legislation, and literature on approaches to treatment without consent (Appelbaum, 1997; Freeman, Pathare, Drew, Funk, & Saraceno, 2005; Roth, Meisel, & Lidz, 1977) were categorized and ranked in order of increasing emphasis on autonomy (Table 2). A legal instrument that incorporated the highest ranked approach along each axis would exceed the requirements of both the WHO and CE guidance statements in terms of respect for autonomy.

### 2.2 Application of the framework

Commonwealth legislation regulating the use of involuntary psychiatric treatment was traced using the University of Cambridge law library and internet searches. Since legislation from Africa was difficult to locate through these sources, we approached the African Association of Psychiatrists for assistance. Care was taken to ensure that the study included legislation enacted over a long period of time (from 1897 to 2007), from countries that were both more and less economically developed, and representing diverse historical and cultural traditions.

To enable meaningful comparison with the guidance, broader criteria for short-term assessment or emergency treatment orders and other legal or quasi-legal instruments regarding involuntary psychiatric treatment (e.g. guardianship legislation, codes of practice and case law) were not included.

All the legislation was read and then coded according to the approach taken to the drafting of the legal tests on each of the framework's five axes. Every Act was given an “autonomy score” (minimum = 6, maximum = 30), calculated by summing the rank score on each axis, plus the types of exclusion criteria. This gives an indication of the weight given to the “respect autonomy” principle in comparison with other considerations, such as patient health or public safety. A high score indicates greater restrictions on the use of involuntary treatment. Compared with a legislation which gains a lower score, therefore, it accords relatively more weight to the principle of respect for patient autonomy and less weight to other principles such as health promotion or public safety.

## 3 Results

Thirty-two examples of legislation were traced; seven of these were from less economically developed countries with a gross national income per capita between $600 and $4180, and 25 from more economically developed countries with a gross national income per capita between $19,990 and $33,630. Whilst mental health legislation from the majority of jurisdictions in the North American, Australasian, Asian and European Commonwealth was traced, it was only possible to trace legislation from three jurisdictions in the Caribbean and one in the African Commonwealth. Two further Asian Acts, nine Caribbean Acts and 11 African Acts were identified but copies of the legislation could not be traced. Nonetheless, the analysis includes legislation from a diverse range of jurisdictions, including some not previously included in this type of comparative analysis.

The results of the coding are shown in Tables 3 and 4. Table 5 shows the variation in approaches to drafting the legal tests according to the age of the legislation and the jurisdiction's level of economic development.

Overall autonomy scores ranged from 8 to 25. The CE recommendations and WHO guidance scored 15 and 16 respectively, and the mean score for all Acts was 15.4, suggesting that a score in the mid teens indicates that legislation achieves a balance between the “respect autonomy” principle and other considerations that is acceptable by current standards.

Over time, mean autonomy scores tend to rise: from 8.7 for the oldest Acts, 15.4 for those enacted in the 1980s and 14.9 in the 1990s, to 18.1 for legislation enacted since 2000. The mean autonomy score in more economically developed countries was 16.4, compared with a mean score of 12 in less economically developed countries (14.5 if the three oldest Acts are excluded). The new legislation for England and Wales is an interesting example as, with a score of 12, it does not follow these trends.

None of the Acts was judged clearly compliant with all of the WHO guidance, although South Africa, Jamaica and five Canadian states fell short only on the stringent requirement for monthly reviews. Four Australian states were judged compliant with all of the CE recommendations. Compliance along each axis is considered in more detail below:

### 3.1 Axis one. Diagnosis

Both the WHO and CE guidance statements suggest including a diagnostic test, based on internationally accepted standards, in legislation. Most legislation appeared compliant. However, nine Acts include very broad diagnostic criteria that, without other safeguards, could lead to non-compliance. The incorporation of a diagnostic test clearly based on internationally accepted standards is most prevalent in legislation enacted during the 1980s. Older legislation uses an “unsound mind” approach, based on judicial rather than medical determination of mental disorder. After the 1980s, there is a trend in countries that are more economically developed to incorporate a very broad definition of mental disorder, tempered in all but two cases by a test of “therapeutic aim” or multiple exclusion criteria.

### 3.2 Axis two. Therapeutic aim

The CE recommendations suggest a requirement that all instances of involuntary admission have a therapeutic aim. Only a minority of Acts (12/32) appear compliant. The incorporation of a test of therapeutic aim is more prevalent both in more economically developed countries and in legislation enacted since 2000.

### 3.3 Axis three. Risk

Both the WHO and CE guidance statements suggest that involuntary admission should be limited to cases where there is a risk of significant or serious deterioration. If judgment is impaired, the WHO guidance recommends that the threshold is lowered to a need for treatment to improve health. Nineteen Acts with a test based on serious deterioration appear compliant with both statements. A further two Acts with a test based on need for treatment plus a capacity test for hospitalization appear compliant with the WHO guidance. Incorporation of a test based on risk to safety or risk of serious deterioration is most prevalent in legislation enacted in the 1980s and 1990s. The majority of legislation enacted since 2000 incorporates a test based on need for treatment. This trend is accompanied by increasing use of capacity tests.

### 3.4 Axis four. Capacity

The WHO guidance suggests a test based on inability to make treatment decisions for the hospitalization of low-risk patients and for the administration of further treatment to all patients. Only eight Acts appear compliant with this standard: Scotland and South Africa set an ability-based capacity test for hospitalization and treatment, while a further six (Jamaica, Pakistan, Yukon, North West Territory, Saskatchewan, Prince Edward Island and Nova Scotia) permit the involuntary hospitalization only of high-risk patients, with an ability-based capacity test for their further treatment. The incorporation of a capacity test is most prevalent in more economically developed countries and in legislation enacted in the 1980s or since 2000.The remaining Acts appear to permit the provision of some form of psychiatric treatment without consent (defined broadly to include hospitalization) to patients who may be able to make treatment decisions. This group includes legislation that permits involuntary treatment for patients who “unreasonably refuse” and legislation that prevents the administration without consent of some (usually invasive or irreversible) forms of psychiatric treatment but permits other forms.

### 3.5 Axis five. Review process

The CE recommendations suggest an automatic independent legal or quasi-legal review of decisions to detain patients, while the WHO guidance suggests intervals of 1 month between reviews. Twenty-two Acts appear compliant with the CE standard; none meets the WHO standard. Incorporation of review procedures is more prevalent in more economically developed countries.

## 4 Discussion

It would not be reasonable to expect that the legislation included in this analysis would all comply with the guidance statements produced by the WHO and the Council of Europe: a) most of the legislation was enacted prior to the publication of the statements, b) the two statements are based on different approaches, and c) only the guidance produced by the WHO was intended to be applicable on all continents. Nevertheless, non-compliance raises an alert that a particular piece of legislation may not adequately safeguard human rights. Our analysis, suggesting considerable variation in the criteria for involuntary admission and treatment, indicates that compliance across the Commonwealth is variable. Possible explanations include differing value perspectives, failure to keep pace with changing attitudes to mental disorder, and variations in the resources available for providing treatment and undertaking law reform. Each of these factors is considered below.

As expected, the analysis suggests that diagnostic thresholds vary with the age of legislation. Acts drafted before effective treatments for mental illness became available use the “unsoundness of mind” approach, emphasizing containment rather than treatment and appearing to reflect attitudes towards men and women with a mental disorder that have now, at least in principle (Thornicroft, 2006), been superceded.

Modern Commonwealth legislation uses a variety of definitions of mental disorder. A definition that is too broad is open to abuse, while one that is too narrow risks excluding people who might benefit from involuntary treatment (Department of Health, 1999). The most recent development is the use of very broad approaches, arguably too broad to provide meaningful guidance. This could be interpreted as leaving the task of diagnosis to those with medical expertise, so that no one in need of treatment is inadvertently excluded, and emphasizing the duty of care over the right to self-determination. However, stringent safeguards such as exclusion criteria would be required to reduce the risk of overly paternalistic interpretations.

The principle of reciprocity has been proposed as one way of balancing respect for self-determination with the duty of public protection (Eastman, 1994). Restrictions of liberty that extend beyond the tariff for a criminal offence may be justified if the person being restricted stands to gain some benefit. A test of therapeutic aim, therefore, provides a means for achieving reciprocity and such a test is found in the CE recommendations. Arguably, a right to treatment is meaningless for a person detained on the basis of a condition that is not amenable to treatment (Harris, 2007) and treatability tests are increasingly being adopted in more economically developed countries. Some jurisdictions have used an alternative method to support reciprocity, the exclusion of “untreatable” conditions. Intellectual disability is a lifelong condition and is the most widespread exclusion criterion found in this study. Elsewhere, a lack of reciprocity may be considered unjust by the courts yet may persist due to lack of resources for treatment (Dhanda, 2005).

The drafting of risk and capacity tests is strongly influenced by value perspectives, which may explain the different approaches adopted by the WHO and the CE. In the 1970s and 80s, increasing emphasis on civil liberties was associated with a move towards stringent risk tests. Restrictions on the freedom of individuals, even those whose judgement was impaired, were only justified in the name of preventing significant harm (Appelbaum, 1997). This was confirmed by this study, with a stringent test found in the majority of modern legislation.

However, the change in approach has not been without its critics. Disorders that compromise insight, volition and mental capacity may prevent those affected from seeking treatment that they might otherwise accept, and untreated psychosis is associated with social exclusion and poor outcomes (Drake, Haley, Akhtar, & Lewis, 2000). The trend towards stringent risk tests has harmed some people with mental disorders who refuse treatment and, as a result, now live marginalized and socially excluded lives (Peele & Chodoff, 1999). This may explain our finding of less stringent risk tests in some of the most recently enacted legislation from Pakistan, South Africa, Scotland, Victoria (Australia) and England and Wales.

When a less stringent test is adopted, an alternative mechanism is required to avoid overly paternalistic treatment of people who are able to make their own decisions Capacity assessment is the mechanism suggested by the WHO and underlies both reason-based approaches that accord absolute respect to rational choices (Kant, 1997 [1785]), and utilitarian and rights-based approaches that reject “hard” paternalism but permit “soft” paternalism when decision-making is impaired (Brock, 1988; Feinberg, 1980; Mill, 1998 [1869]). In contrast with the 1980s and 1990s, when a “stringent risk test-no capacity test” approach was the most frequent approach, we found that, in legislation enacted since 2000, 50% (5/10) of the Acts we identified had adopted some form of capacity threshold for hospitalization. It is interesting to note that both the “stringent risk test” and the “capacity-based” approaches are legitimate alternatives for the regulation of involuntary treatment within some jurisdictions. For example, most Australian States have a stringent risk test and no capacity test in their mental health legislation but all allow health-care decisions to be made on behalf of incapacitated adults under guardianship legislation (such legislation was not included in our analysis, which was limited to more specific mental health legislation), which uses a low risk threshold based on best interests and an ability-based capacity test. Both types of legislation generate similar autonomy scores.

Nonetheless, the strongly paternalistic combination of a low risk threshold and low incapacity threshold is still being adopted. This may be due to the use of the outcome-based approach to capacity, permitting compulsory treatment in cases of unreasonable refusal (Roth et al., 1977). Such an approach is explicitly adopted in two Australian states (Queensland and the Northern Territory) and implicit in an approach taken elsewhere when competent refusals carry more weight when invasive or burdensome treatment is concerned (for example, in England and Wales where, according to ss57–58 of the *Mental Health Act 1983*, competent refusals of highly invasive treatment must be respected and competent refusals of burdensome treatment can only be overridden with the support of an independent second opinion). Although this outcome-based approach is sanctioned in UN Resolution 46/119 (principle 11, paragraph 6b), it has been criticized as tending to result in individuals being judged to lack decision-making capacity if they disagree with their doctor (Gunn, 1994), making the “ability” standard preferable.

Another possible reason, relevant to legislation that makes no reference to capacity, is a tacit application of a status-based approach (Roth et al., 1977), based upon the assumption that all members of a particular group (in this case, people diagnosed with a mental disorder) lack decision-making capacity. This assumption is erroneous: empirical studies indicate that the majority of psychiatric in-patients are capable of making treatment decisions (for example, Bellhouse, Holland, Clare, Watson, & Gunn, 2003a; Wong et al., 2000) and this capacity can be reliably assessed using a checklist derived from legal definitions (Bellhouse, Holland, Clare, Watson, & Gunn, 2003b).

Finally, both the guidance statements require regular independent reviews to ensure that decisions to detain and treat are only made when patients meet the threshold criteria, meaning that self-determination is only limited when this is shown to be necessary. Review mechanisms provide a more robust safeguard against inappropriate detention than appeal processes, as they are not initiated by the patient, who may be subject to undue influence or lack resources to take a case to court. However, reviews can be costly in terms of financial resources and the time of clinicians, possibly compromising clinical care, and so may not be given high priority when patients are not objecting to treatment. This could explain the finding that the majority of low resource countries in this study had no automatic review process. Given evidence that “there is a basic problem in every country in the world: that the supply of treatment and care is less or far less than the actual need” (Thornicroft, 2006 p267), such costs may also contribute to the lack of compliance with the stringent WHO guidance on review mechanisms that we found even in those jurisdictions that are more economically developed.

Data on rates of detention under different legal systems suggest that factors other than legal criteria influence decisions to detain a patient (Appelbaum, 1997; Zinkler & Priebe, 2002). This study does not does not show how closely the legislation is adhered to in practice in any given country. Further research, of the type carried out by in the UK by Quirk (Quirk, Lelliot, Audini, & Buston, 2000) and in the USA by Hoge (Hoge et al., 1997, 1998), is needed to investigate ways in which different legal regulations are interpreted in practice and affect the experiences of people with a mental disorder.

In summary, differences in value judgments regarding underlying principles, attitudes to mental disorder, and resource availability may all contribute to variation in criteria for involuntary treatment. Ideally, legislation should accommodate heterogeneous values, including those of patients who may value their liberty over their health or vice versa (Fulford, 2004). It should not be based upon attitudes contradicted by research evidence. Where resources are an issue, richer nations could assist to ensure that the human rights of people of all nations are respected.

Following from the findings reported in this paper, a combination of examples of good practice from seven jurisdictions with relatively recent mental health legislation were used to produce suggested threshold criteria for the protection of people who may be subject to involuntary psychiatric treatment. These suggestions reflect current standards, described by the WHO and CE guidance statements, and trends, demonstrated by the data presented in this paper, and are informed by the ethical theory we have outlined in the Introduction.

1. A diagnostic test based on functional impairment and symptoms, informed by the ICD-10. This was the most prevalent approach found in this study. It is based upon a social model of mental disorder and focuses on the difficulties being experienced by the patient. Although, in comparison with the syndrome-based approach, it is more difficult to operationalise, it is transparent to people without medical training (Freeman et al., 2005). It can be structured to cover only problems associated with serious mental disorder, such as those listed in ICD-10 (World Health Organization, 1992), avoiding the potential problem of over-inclusiveness leading to abuse. An example is used in Northern Territory, Australia: “a condition that seriously impairs, either temporarily or permanently, the mental functioning of a person in one or more areas of thought, mood, volition, perception, orientation or memory and is characterized (a) by the presence of at least one of the following symptoms: delusions, hallucinations, serious disorders of the stream of thought, serious disorders of thought form, serious disturbances of mood or (b) by sustained or repeated irrational behaviour that may be taken to indicate the presence of at least one of the symptoms referred to in (a).”
2. Exclusion criteria for the prevention of the misuse of psychiatry as a means of political or social control. The use of ethnic, political, religious or cultural status as an exclusion is noticeable in the legislation of South Africa, Australia and New Zealand, all of which have an unfortunate history of oppression of indigenous peoples. An example is used in New Zealand, where use of involuntary treatment solely for reasons including “political, religious or cultural beliefs…sexual preferences…criminal or delinquent behaviour…” is not allowed. Additional guidelines suggest that the Act should not be applied in cases of psychosexual disorder or anti-social personality disorder if an individual chooses to act and can take responsibility for the outcome, linking self-determination with personal responsibility.
3. A treatability test, requiring that treatment likely to alleviate the effects of the disorder, or prevent it worsening, is available for the patient. A treatability test is a robust means of achieving reciprocity (Eastman, 1994) without the need for constant revision of a list of “untreatable” exclusions. Personality disorders, sometimes excluded from use as a justification for involuntary treatment, are widely believed to be lifelong conditions although recent evidence challenges this assumption (Tyrer et al., 2007). The suggested approach is more stringent than the current Council of Europe standard (therapeutic intent), which has been criticized because reciprocity is not achieved unless the detained person receives a benefit (Harris, 2007). An example is used in Scotland: “treatment which would be likely to prevent the mental disorder worsening or alleviate any of the symptoms, or effects, of the disorder, is available for the patient”*.*
4. A risk test based on welfare interests. A relatively low threshold reduces the risk of disability associated with long duration of untreated psychosis, and is consistent with WHO standards if combined with a suitable capacity test. An example of the approach is used in both the Pakistani and English legislation: treatment “is necessary for the health … of the patient”.
5. A capacity test based on decision-making ability. In many common-law jurisdictions, the status and outcome-based approaches to evaluating capacity to consent to treatment for physical ill-health have been replaced by a test of ability to understand relevant information (Kennedy & Grubb, 2000). When mental health legislation preserves an exception to this general principle for psychiatric treatment, unjustified discrimination may occur. The ability approach can be found in the South African legislation which requires that the patient must not be given involuntary treatment unless he or she is “incapable of making an informed decision on the need for the care, treatment and rehabilitation services*.*” In Queensland, Australia, capacity to make treatment decisions is defined as: “capable of understanding the nature and effect of decisions…and freely and voluntarily making decisions…and communicating the decisions in some way.”
6. Special provisions for the treatment of people who pose a high risk to others. Following the principle of utility (Mill, 2001 [1861]), one exception to the approach to risk and capacity described above would be acceptable—the involuntary treatment of people who have the capacity to refuse treatment, but represent a significant risk to others that could be reduced with such treatment. To avoid discrimination towards people with mental rather than physical disorders, a limited form of the narrow safety approach that excludes risk to self would be needed. This is consistent with the approach of many common-law jurisdictions to competent refusals of treatment for physical illness; they are not overridden even if this will lead to the patient's death (Kennedy & Grubb, 2000). The exception is statutory provisions to override refusals of treatment for physical illness that place other people at significant risk of harm, for example through the spread of infectious disease. The use of risk of serious harm as a justification for involuntary hospitalization can be problematic due to the fallibility of risk assessment. Serious assault by people with mental disorder is rare and the false positive rate is likely to be high (Szmukler, 2003; Taylor & Gunn, 1999). A safeguard against unnecessary restriction of liberty could involve making this type of detention part of the criminal justice system, applied to people who would otherwise serve a prison sentence. An example is found in Scotland, where an order for lifelong restriction (OLR) can be imposed after risk assessment of a person convicted of a violent offence, combined with a hospital order in cases of mental disorder (Darjee & Crichton, 2002). After a tariff for the offence had been served, release is dependent on the level of continuing risk of serious recidivism. Offenders are released on licence, subject to conditions intended to reduce risk and recalled if those conditions are not met. It is anticipated that most OLRs will be applied to people with personality disorders (Tuddenham & Baird, 2007).
7. Regular tribunal reviews of patients made subject to involuntary treatment. An example is the system used in Northern Territory, Australia. All involuntary admissions and orders for involuntary outpatient treatment must be reviewed by tribunal within 1 week. Thereafter, involuntary admissions must be reviewed by tribunal every 3 months and community management orders reviewed every 6 months. Patients, and persons with a legitimate interest in their wellbeing, may appeal against involuntary admissions and community management orders. Voluntary admissions must be reviewed by tribunal every 6 months to ensure that the patient is capable of giving informed consent.

Mental health legislation based on these seven features would, according to our analysis, have an “autonomy score” of 20. This is comparable to the scores achieved through the use of the WHO and CE guidance (16 and 15 respectively) and should therefore be sufficient to protect the human rights of men and women with a mental disorder without compromising the provision of treatment to those in need.

One of the most striking findings of this study results from recent law reform in the UK. Before 2003, there was little difference between English and Scottish criteria for involuntary psychiatric treatment. Since then, approaches to diagnostic, treatability and capacity tests have diverged. *The Mental Health (Care and Treatment) (Scotland) Act 2003* has an autonomy score of 22. In stark contrast, the *Mental Health Acts 1983* and *2007*, which operate in England and Wales, have an autonomy score of 12. It remains to be seen whether these changes will translate into differences in clinical outcomes.

## Figures and Tables

**Table 1:** Two models for the protection of human rights through legislation for the regulation of involuntary psychiatric treatment.

Legal test	WHO guidance	CE recommendations
Diagnosis	“Qualified mental health professionals… should determine that the person in question has a mental disorder.” Para 3.1.1	“Applies to persons with mental disorder defined in accordance with internationally accepted medical standards. Lack of adaption to the moral, social, political or other values of a society, of itself, should not be considered a mental disorder.” Art 2.
	“Evidence of a mental disorder of specified severity as defined by internationally accepted standards.” Para 3.1.4	
Therapeutic aim		“A person may be subject to involuntary placement only if…the placement includes a therapeutic purpose.” Art 17.
		“Therapeutic purposes include prevention, diagnosis, control or cure of the disorder, and rehabilitation… Treatment may include measures to improve the social dimension of a person's life.” Art 2
Risk	“They should be convinced [of]… a high probability of immediate or imminent harm to this person or other persons, or, in the case of a person whose mental disorder is severe and whose judgment is impaired, that failure to admit or detain that person would probably lead to a serious deterioration in his or her condition or would prevent appropriate treatment.” Para 3.1.1	“A significant risk of serious harm to his or her health or to other persons.” Arts 17 & 18
Capacity	“Competence to give consent…refers…to the capacity to understand the purpose, nature and likely effects of a particular treatment” Para 3.1.8	
	“Legislation may allow treatment to proceed without informed consent…if a person…is found to be lacking competence.” Para 3.1.3	
Review process	“Legislative provision for automatic review mechanisms in all cases of involuntary admission and treatment…Reviews should take place at reasonable intervals e.g. no longer than monthly…They should be conducted by an independent regulatory body with legal or quasi-legal status for the enforcement of good practice.” Para 3.1.7	“Right to appeal against a decision; to have the lawfulness of the measure, or its continuing application, reviewed by a court at reasonable intervals; to be heard in person or through a personal advocate or representative at such reviews or appeals.” Art 25

**Table 2:** Framework for the comparative analysis of legislation.

**Axis 1. Diagnosis**
Level 1. No definition of mental disorder in the legislation, and no standard set for determining its presence.
Level 2. “Unsoundness of mind” approaches, determined by legal professionals and emphasize a perceived need for control or containment.
Level 3. “Disability” approaches—based on the presence of phenomena that impair mental functioning.
Level 4. Broad “disorder” approaches—based on the diagnosis of particular syndromes or classes of syndrome.
Level 5. Narrow “disorder” approaches—based on an internationally recognized system of classification e.g. ICD-10 or DSM-IV.
**Supplement to axis 1—exclusion criteria**
If the legislation excluded conditions from being considered grounds for involuntary treatment, these were also noted and classified as follows:
Group a) ethnicity; religious, political, cultural or philosophical beliefs or practices.
Group b) criminal, irresponsible or antisocial behaviour.
Group c) sexual preference, identity or practices.
Group d) misuse of alcohol or drugs.
Group e) intellectual disability.
Group f) personality disorder (may be limited to cluster B or to anti-social personality disorder).
**Axis 2. Therapeutic aim**
Level 1. No therapeutic intent required —detention justified by public interest.
Level 2. Requirement for therapeutic intent for involuntary admission.
Level 3. Requirement that treatment for the condition is available.
Level 4. Treatment must be likely to alleviate the condition or prevent deterioration.
**Axis 3. Risk**
Level 1 Detention permitted when degree of risk is unknown.
Level 2. Broad “health” approaches—detention needed to bring about an improvement in health or ability to function.
Level 3. Narrow “health” approaches—detention needed to prevent deterioration.
Level 4. Broad “safety” approaches—detention needed to prevent a significant or serious deterioration or psychological harm to the patient or others.
Level 5. Narrow “safety” approaches — detention needed to prevent immediate or imminent physical harm to the patient or others.
**Axis 4. Capacity (a. for hospitalization and b. for other treatment)**
Level 1. No capacity threshold—treatment permitted without a capacity assessment or when the patient is able to make a treatment decision.
Level 2. Outcome approaches—the patient makes an irrational choice or the outcome of the patient's treatment decision is deemed unreasonable.
Level 3. Ability approaches—the patient is found to lack the ability to make the treatment decision.
**Axis 5. Review process**
Level 1. No review or appeal process.
Level 2. Right of appeal but no automatic independent legal review.
Level 3. Regular automatic independent legal review.
Level 4. Monthly automatic independent legal review.

**Table 3:** Results of the coding process in areas with gross national income per capita US$ 600-4 180.

Origin of framework	Date	Diagnosis	Exclusion criteria abcdef^1^	Treatability	Risk	Incapacity (admission)	Incapacity (treatment)	Review process	Autonomy rating (6–30)
		+++++		++++	+++++	+++	+++	++++	
**WHO**	**2003**	+++		+	++++/++	+/+++	+++	++++	**16**
**Council of Europe**	**2004**	+++	a	++	++++	+	+	+++	**15**
Sri Lanka	1873	++		+	++	++	++	+	10
Grenada	1895	++		+	+	+	+	++	8
St Lucia	1895	++		+	+	+	+	++	8
India	1987	++++	e	+	++	+	+	++	12
Jamaica	1999	+++		+	++	+	+++	+++	13
Pakistan	2001	++++	cd	+	++	+	+++	++	15
South Africa	2002	+++++	a	+	++	+++	+++	+++	18

Number of + signs indicates the level of the framework the legislation is judged to have reached, maximum indicated at top of column.

1 a) ethnicity; religious, political, cultural or philosophical beliefs or practices, b) criminal, irresponsible or antisocial behaviour, c) sexual preference, identity or practices, d) misuse of alcohol or drugs, e) intellectual disability, f) personality disorder (may be limited to cluster B or to anti-social PD).

**Table 4:** Results of the coding process in areas with gross national income per capita US9 990–33 630.

Origin of framework	Date enacted	Diagnosis	Exclusion criteria	Treatability	Risk	Incapacity (admission)	Incapacity (treatment)	Review process	Autonomy rating (6–30)
		+++++	abcdef^2^	++++	+++++	+++	+++	++++	
**WHO**	**2003**	+++		+	++++/++	+/+++	+++	++++	**16**
**Council of Europe**	**2004**	+++	a	++	++++	+	+	+++	**15**
England & Wales	1983	++++	cd	++++^3^/+	++	+	+	+++	15
Northern Ireland	1986	+++		+	+++++	+	+	++	13
Saskatchewan (Canada)	1986	+++		++	++++	+++	+++	+++	18
Northwest Territories (Canada)	1988	+++	e	+	+++++	+	+++	+++	17
Prince Edward Island (Canada)	1988	+++	e	+	+++++	+	+++	+++	17
Nova Scotia (Canada)	1989	+++		+	+++++	+	+++	+++	16
Newfoundland (Canada)	1990	+		+	+++++	+	+	++	11
New South Wales (Australia)^4^	1990	+	abcde	+	++++	+	+	+++	16
South Australia	1993	+		+++	++++	+	+	+++	13
Australian Capital Territory	1994	+++	abcde	++++	++++	+	+	+++	21
New Brunswick (Canada)	1994	+++	e	+	++++	+	+	+++	14
British Columbia (Canada)	1996	+++		+	+++	+	+	++	11
Tasmania (Australia)	1996	+++	ab e	+++	++++	+	+	+++	18
Western Australia	1996	+++	abcde	+++	++	+	+	+++	18
Manitoba (Canada)	1998	+++	e	++	++++	+	+	+++	15
Quebec (Canada)	1998	+		+	++++	+	+	++	10
New Zealand	1999	+++	abcdef	+	++++	+	+	+++	19
Alberta (Canada)	2000	+++		++++	+++++	+	+	+++	17
Ontario (Canada)	2000	+		+/++++	+++++/++++	+/+++^5^	+++	+++	16
Queensland (Australia)	2000	+++	abcde	+++	++++	++	++	+++	21
Yukon (Canada)	2002	+++		+	+++++	+	+++	+++	16
Scotland	2003	++++	bcd	++++	+++	+++	+++	++	22
Victoria (Australia)	2003	+++	abcdef	+++	++	+	+	+++	19
Northern Territory (Australia)	2005	+++++	abcdef	+++	++++	++	++	+++	25
England & Wales	2007	+	de	++	++	+	+	+++	12

2 a) ethnicity; religious, political, cultural or philosophical beliefs or practices, b) criminal, irresponsible or antisocial behaviour, c) sexual preference, identity or practices, d) misuse of alcohol or drugs, e) intellectual disability, f) personality disorder (may be limited to cluster B or to anti-social PD).

3 Treatability test only applies to diagnostic categories roughly equivalent to personality disorder and mild to moderate intellectual disability.

4 NSW legislation has been updated since this analysis was conducted, with new legislation enacted in 2007.

5 Thresholds for treatability and incapacity vary with level of risk.

**Table 5:** Variation in approaches to threshold setting and compliance with standards a) over time and b) according to gross national income per capita.

Legal test	Modal category^6^				Percentage compliance with WHO and CE standards				Modal category^1^		Percentage compliance with WHO and CE standards	
	old	80s	90s	00s	Old	80s	90s	00s	Low income	High income	Low income	High income
Diagnosis	Unsound mind	Disability	Disability	Disability	0 (0/3)	100(7/7)	67(8/12)	80(8/10)	Unsound mind	Disability	57(4/7)	76(19/25)
	++	+++	+++	+++					++	+++		
Treatability	None +	None +	None +	Effective for condition/effective for individual^7^ +++/++++	100(3/3)	100 (7/7)	100(12/12)	100(10/10)	None +	None +	100(3/3)	100(25/25)
					0 (0/3)	14 (1/7)	42 (5/12)	60 (6/10)			0 (0/3)	48 (12/25)
Risk	None +	Narrow safety +++++	Broad safety ++++	Broad health ++	0 (0/3)	71 (5/7)	75 (9/12)	70 (7/10)	Broad health ++	Broad safety ++++	14 (1/7)	80 (20/25)
					0 (0/3)	71 (5/7)	75 (9/12)	50 (5/10)			0 (0/7)	76 (19/25)
Incapacity (admission)	None +	None +	None +	None +	0 (0/3)	71 (5/7)	75 (9/12)	70 (7/10)	None +	None +	0 (0/7)	80 (20/25)
					100 (3/3)	100 (7/7)	100(12/12)	100(10/10)			100 (7/7)	100(25/25)
Incapacity (treatment)	None +	Ability +++	None +	Ability +++	0 (0/3)	57 (4/7)	1 (1/12)	50 (5/10)	None +	None/Ability^8^ +/+++	0 (0/7)	28 (7/25)
					100 (3/3)	100 (7/7)	100(12/12)	100(10/10)			100 (7/7)	100(25/25)
Review procedure	Appeal ++	Review +++	Review +++	Review +++	0 (0/3)	0 (0/7)	0 (0/12)	0 (0/10)	Appeal ++	Review +++	0 (0/7)	0 (0/25)
					0 (0/3)	71 (5/7)	75 (9/12)	80 (8/10)			29 (2/7)	80 (20/25)

6 Refer to Table 2 for the definitions of each category.

7 Bimodal distribution.

8 Bimodal distribution.
